# Tumor metabolome remolded by low dose mitochondrial uncoupler elicites robust CD8^+^ T cell response

**DOI:** 10.1038/s41420-025-02584-9

**Published:** 2025-07-01

**Authors:** Xiaoxiao Jiang, Zhijin Fan, Zhenzhen Zhang, Fanchu Zeng, Tong Sun, Yuchen Li, Guojia Huang, Liming Nie

**Affiliations:** 1 Medical Research Institute, Guangdong Provincial People’s Hospital (Guangdong Academy of Medical Sciences), Southern Medical University, Guangzhou, China; 2 School of Medicine, South China University of Technology, Guangzhou, China; 3 Key Laboratory of Brain, Cognition and Education Sciences, Ministry of Education, China; Institute for Brain Research and Rehabilitation, and Guangdong Key Laboratory of Mental Health and Cognitive Science, South China Normal University, Guangzhou, China

**Keywords:** Cancer metabolism, Tumour immunology

## Abstract

Tumor cells balance ATP production and carbon skeleton synthesis by flexibly altering catabolic pathways to sustain their significant growth advantage. Uncouplers have shown potential for tumor suppression by converting chemical energy from catabolism into heat. However, their use may be limited due to indiscriminate metabolic interference in both tumor and normal cells, as well as the uncertainty surrounding their effects on the immune microenvironment. Herein, we found that low-dose uncoupler BAM15 promoted AMPK, AKT signaling, and the TCA cycle without increasing cell proliferation or inducing cell death in vitro, suggesting an increase in futile cycling. Intratumoral injection of 50 ng/mL BAM15 accelerated catabolic processes while inhibiting anabolic pathways, resulting in a metabolomic reshaping with increased levels of linoleic acid, C5DC, and others. These changes were shown to enhance tumor-killing effects by T cells. To reduce side effects on normal tissues and improve tumor retention, BAM15 was targeted for delivery by loading it into TCVs. This TCV-BAM15 treatment significantly increased CD8+ T cell counts and granzyme B levels. Our findings highlight a previously unrecognized therapeutic effect and signaling mechanism of low-dose BAM15 treatment in tumors. We propose that this novel strategy holds promise as a tumor immunity therapy with fewer adverse effects compared to free uncoupling drugs at high concentrations.

## Introduction

Aerobic glycolysis generates ATP and carbon skeletons essential for cell proliferation and has long been viewed as the primary metabolic pathway for tumor growth. However, advancements in metabolic flow analysis have shown that the TCA cycle, which produces ATP more efficiently, still operates alongside the Warburg effect [1]. Additionally, the ability of tumor cells to flexibly switch between metabolic pathways, including the pentose phosphate pathway (PPP), the one-carbon pathway, and the glutamine pathway, facilitates their rapid proliferation even in nutrient-poor environments. This flexibility helps maintain an ideal ATP to carbon skeleton ratio and fosters a growth-supportive immune microenvironment. In theory, targeting this optimal ATP/carbon ratio could hinder tumor progression. Dietary restrictions and pharmacological strategies have emerged to disrupt tumor metabolism [2–5]. The loss of tumorigenic mutations related to energy production, macromolecular synthesis, and redox regulation has been shown to slow tumor advancement [6]. However, the intricacy and adaptability of metabolic networks [7] highlight potential vulnerabilities for long-term metabolic intervention benefits.

The reprogramming of tumor-infiltrating immune cells to adopt pro-tumor functions represents another hallmark of cancer. Accumulation of lactic acid leads to the expression of tumor-promoting genes in macrophages [8]. Glutamine supports tumor immune escape [9], and blocking glutamine could reverse tumor immune evasion [10]. Elevated levels of succinate [11, 12] and fumarate [13] derived from tumors impair the anti-tumor activity of CD8^+^ T cells. Linoleic acid has been found to prevent T-cell exhaustion within tumor environments. The ability of metabolic enzymes and metabolites to modulate tumor immunity complicates the deregulation of tumor metabolism.

Uncouplers, which can convert chemical energy to heat by transporting protons across the inner mitochondrial membrane independent of ATP synthase, emerged as a promising avenue for tumor metabolic therapy [14]. Various protonophores, such as 2,4-dinitrophenol (DNP) [15], FCCP [16], CCCP [17], and BAM15 [18], have been shown to directly increase mitochondrial metabolic stress [19, 20]. BAM15 has been demonstrated not to depolarize the plasma membrane [21] and possesses a strong safety profile [22], indicating its potential as a suitable pharmacological mitochondrial uncoupler for in vivo applications. Studies have shown that BAM15 can inhibit tumor cell growth at doses ranging from 2 to 10 μM [23, 24]; However, high-concentration BAM15 may cause significant weight loss due to its non-selective metabolic acceleration of both tumor and normal cells, potentially leading to unfavorable clinical outcomes [25].

**Fig. 1:**
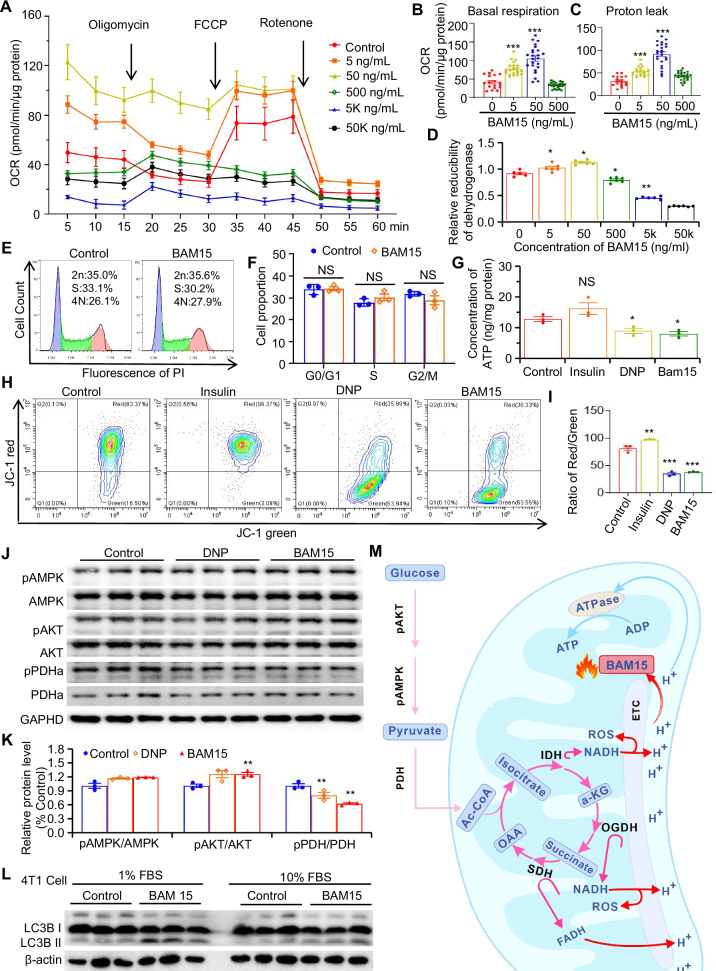
Low dose of BAM15 increases futile energy expenditure in 4T1 tumor cells. **A** Oxygen consumption rates of 4T1 tumor cells after 24 h of exposure to BAM15 at various concentrations (0, 5, 50, 500, 5, and 50 k ng/mL). **B** Changes in basal oxygen consumption rates under the described conditions of BAM15. **C** Changes in proton leak oxygen consumption rates under the described conditions of BAM15. **D** Mitochondrial dehydrogenase activity detection by using WST-8 at various dosages of BAM15 (*n* = 6). **E** Cell cycle analysis with or without 50 ng/mL BAM15 (*n* = 3). **F** Quantification of each phase by PI staining. **G**. ATP concentration measurement under DMSO, insulin, BAM15, and DNP treatment in 4T1 cells (*n* = 5). ***H*** Representative flow cytometry plot and **I** quantification of JC-1 fluorescence in 4T1 cells exposed to insulin, BAM15, or DNP for 24 h (n = 3). **J** Phosphoration of AMPK, AKT, and PDHa was measured via western blotting. **K** Quantification of the ratio of phospho-protein/total protein shown in (**J**). **L** Western blot analysis of protein levels under BAM15 treatment in 4T1 cell within 1% or 10% FBS. **M** Schematic diagram of how BAM15 promotes energy metabolism. Group differences were analyzed using two-tailed *t*-tests. Data were normalized to the control group and are presented as mean ± SEM (*n* ≥ 3). Statistical significance was defined as ***P < 0.001, *P < 0.01, and *P < 0.05 versus the control group; NS indicates nonsignificant differences.

**Fig. 2:**
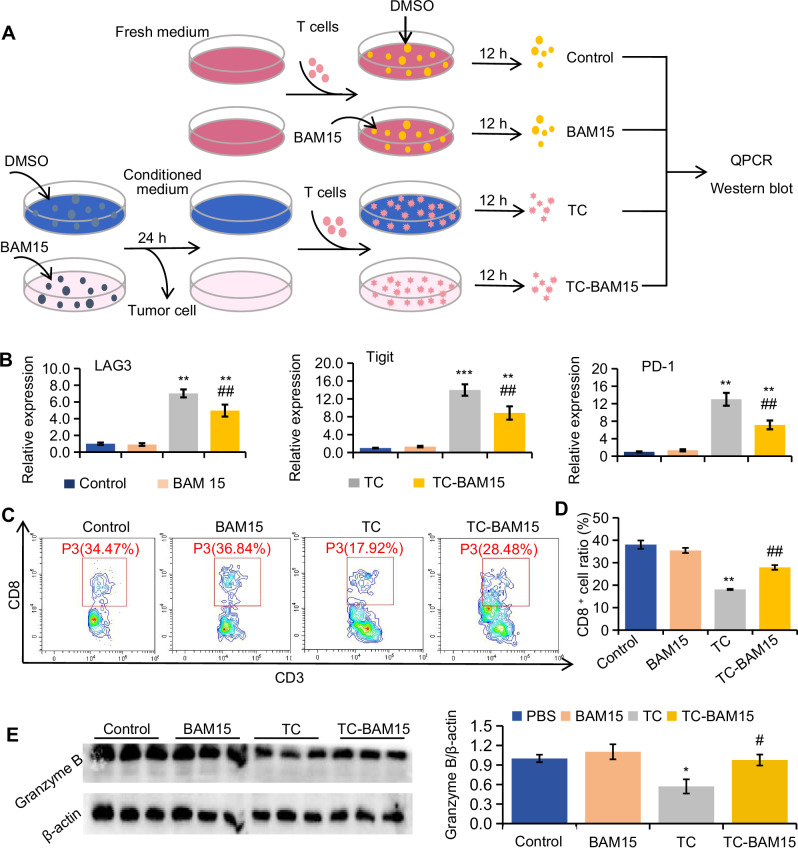
Tumor secretions reshape by BAM15 directly increase T cell response. **A** Schematic of the cell experimental design. **B** Detection of the expression of genes related to exhausted T cells via QPCR. **C** Ratio detection and **D** Quantitative analysis of CD8^+^ T cells by FACS. **E** Western blot analysis of granzyme B in T cells subjected to the indicated treatments. Group differences were assessed by two-tailed *t*-tests with data normalized to controls (mean ± SEM, *n* ≥ 3). Significance levels: ***P < 0.001, **P < 0.01, *P < 0.05 vs control; ##*P* < 0.01, #*P* < 0.05 vs TC; NS not significant.

**Fig. 3:**
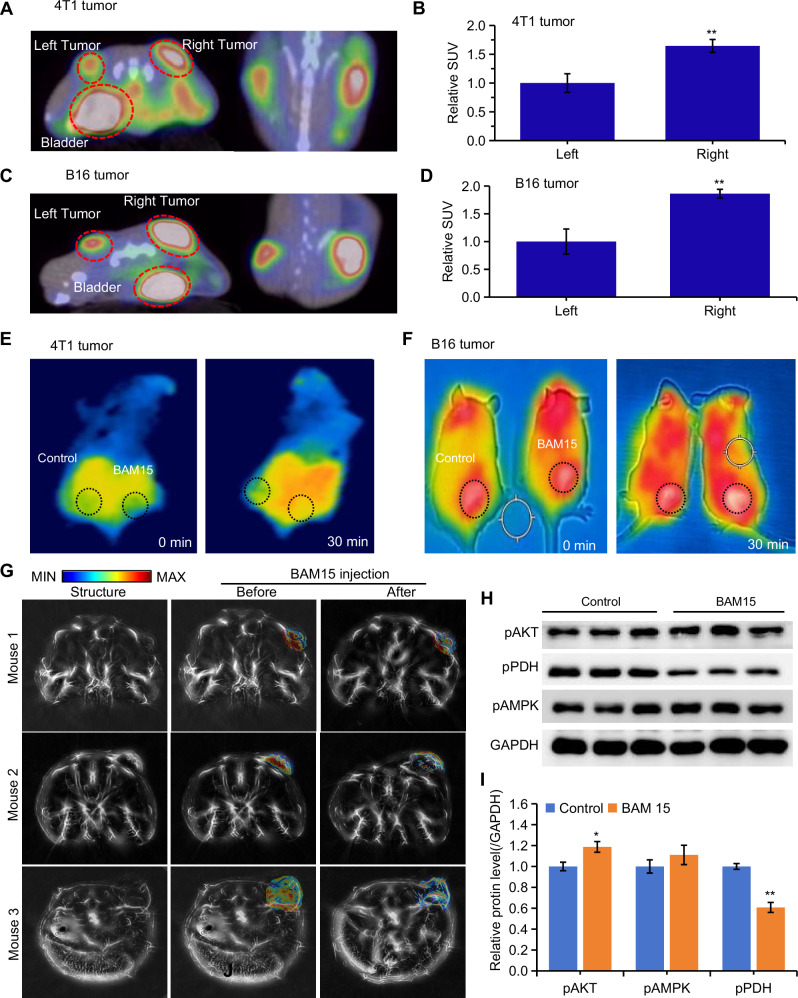
Intratumoral injection of BAM15 increases oxidative metabolism. **A** ^18^FDG-PET/CT images of glucose uptake and distribution in bilateral mastoncus (4T1 cell tumor)-bearing mice 20 min after injection with PBS (left) or BAM15 (right). **B** Intensities of the regions of interest in the PET/CT images from (**A**). **C** ^18^FDG-PET/CT images of glucose uptake and distribution in the melanoma (B16 cell tumor) 20 min after injection with the indicated agents. **D** Intensities of the regions of interest in PET/CT images from (**C**). Surface temperature detection of mastoncus (**E**) and melanoma (**F**) before and 30 min after BAM15 intratumor injection. **G** Dual-wavelength photoacoustic computed tomography detection of mastococcal oxygen saturation (sO_2_) before and 10 min after BAM15 injection. **H** Phosphoration of AMPK, AKT, and PDHa was measured via western blotting. **I** Quantification of the ratio of phospho-protein/total protein shown in (**H**). Group differences were assessed by two-tailed *t*-tests with data normalized to controls (mean ± SEM, *n* ≥ 3). Significance levels: **P < 0.01, *P < 0.05 vs control.

**Fig. 4:**
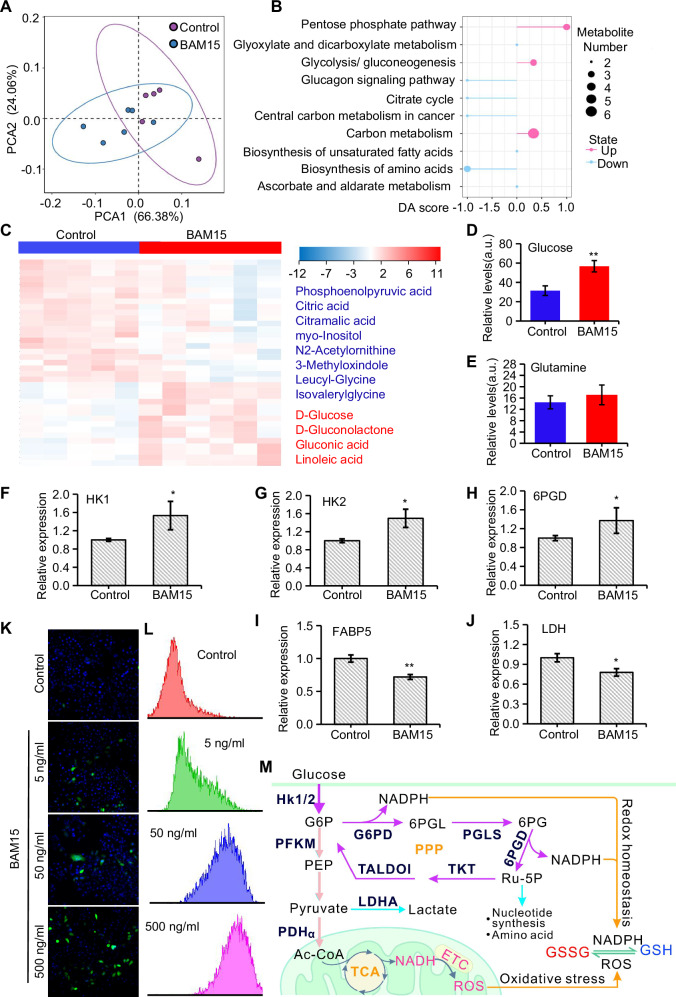
BAM15 treatment promotes catabolism in tumor tissue. **A** Principal component analysis of metabolites in tumors from different groups. **B** KEGG pathway enrichment analysis of tumors from different groups. **C** Heatmap showing the relative abundances of metabolites in different groups. **D** Statistical analysis of the abundance of D-glucose. **E** Statistical analysis of the abundance of L-glutamine. **F**–**J** Detection of the expression of key enzymes of classical metabolic pathways in 4T1 cells. **K** ROS staining of 4T1 cells treated with serial dosages of BAM15. **L** FACS analysis of ROS production in 4T1 cells treated with serial dosages of BAM15. **M**. Characteristics of the reprogrammed metabolic pattern of tumors induced by BAM15. Group differences were assessed by two-tailed *t*-tests with data normalized to controls (mean ± SEM, *n* ≥ 3). Significance levels: **P < 0.01, *P < 0.05 vs control.

**Fig. 5:**
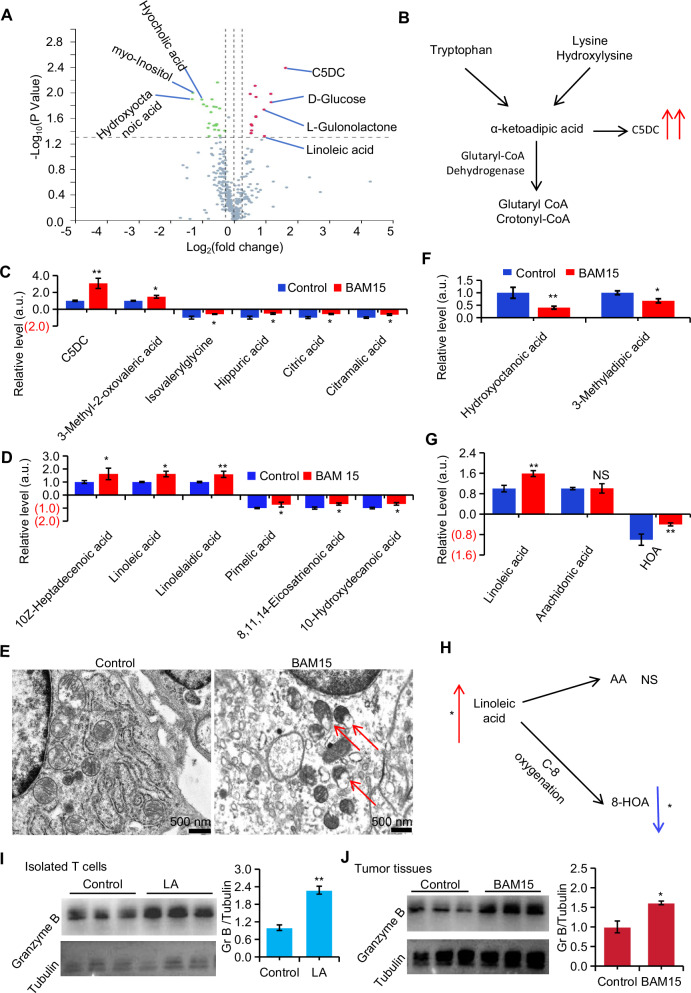
BAM15 enhances T cell cytotoxicity partly dependent on mitochondrial metabolites accumulation. **A** Volcano plots of annotated polar metabolites. **B** Mitochondrial metabolic pathways associated with C5DC accumulation. **C** Content analysis of representative metabolites associated with mitochondrial enzymes. **D** Content analysis of metabolites associated with fatty acid oxidation. **E** Mitochondria morphology by transmission electron microscope with or without BAM15. **F** Content analysis of representative free fatty acids. **G** Content analysis of linoleic acid metabolism-related products. **H** Metabolic atlas of linoleic acid. **I** Western blot analysis of granzyme B in T cells treated with LA. **J** Western blot analysis of granzyme B tumors in with or without BAM15. Group differences were analyzed using two-tailed *t*-tests. Data were normalized to the control group and are presented as mean ± SEM (*n* ≥ 3). Statistical significance was defined as ***P < 0.001, *P < 0.01, and *P < 0.05 versus the control group; NS indicates nonsignificant differences.

**Fig. 6:**
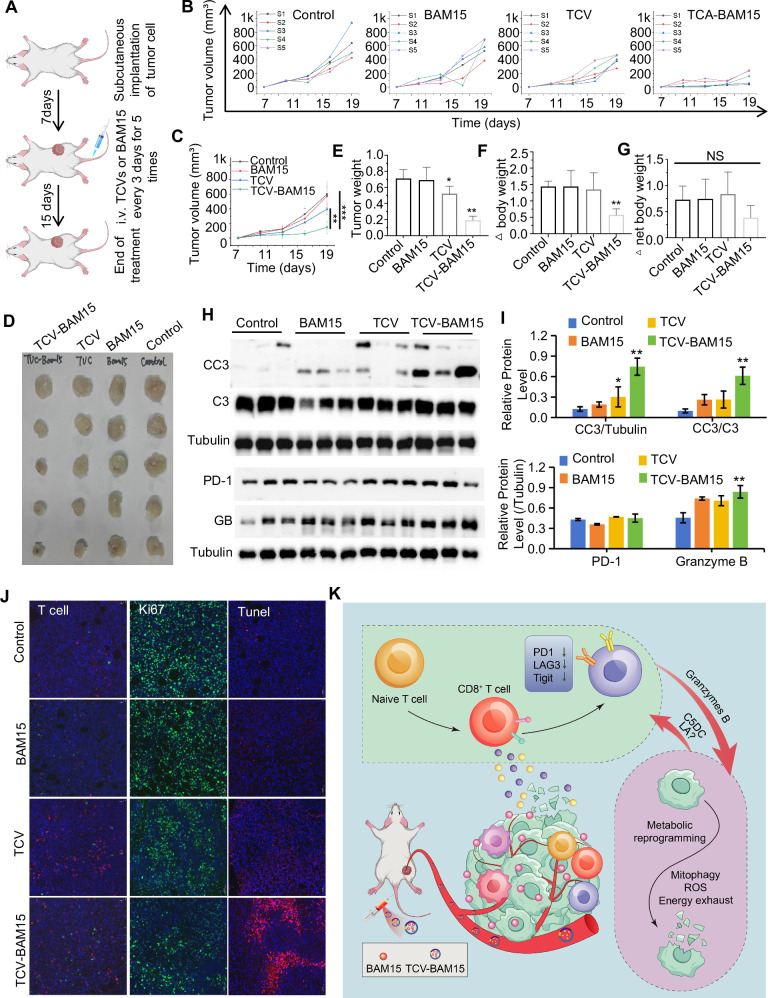
TCV-BAM15 effectively inhibits tumor growth. **A** Flow chart of the animal experiments. **B** Tumor volume growth curves of tumor-bearing mice. **C** Statistical analysis of tumor size in the 4 treatment groups. **D** Images of the tumors in each group. **E** Tumor weights of the mice. **F** Changes in the body weight of the mice. **G** Net weight change after tumor removal. **H** The expression of cleaved caspase-3, PD-1, and GrB in each group was detected by western blot. **I** The intensities of the indicated protein bands were determined via ImageJ and normalized to those of Tubulin **J** TUNEL, ki67, and CD4/CD8 in tumors were detected via tumor biopsy. **K** Cartoon showing how TCV-BAM15 works against cancer. Statistical analysis was performed using one-way ANOVA for **C** and two-tailed *t*-tests for other group comparisons. All data were normalized to the control group and expressed as mean ± SEM (*n* ≥ 3). The statistical significance criteria were defined as: ***P < 0.001, **P < 0.01, *P < 0.05 versus control group; NS indicates nonsignificant differences.

In our research, we found that a low concentration of BAM15 (50 ng/mL, 147 nM) effectively uncoupled mitochondrial metabolism in tumor cells (Fig. 1) and initiated compensatory metabolic pathways activation. The intratumoral administration of BAM15 at 50 ng/mL favored oxidative metabolism in tumors (Figs. 2–4), resulting in changes to the metabolite profile (Fig. 5). These altered metabolites enhance the tumor-killing effects of T-cells, both directly and indirectly, by alleviating T-cell exhaustion and increasing granzyme B secretion. Given the rapid clearance of BAM15 in vivo [22] and its minimal impact on tumor therapy from low-dose tail vein injections (Fig. 6), tumor-infiltrating T lymphocyte-derived cellular vesicles (TCVs) were engineered as tumor-targeted delivery vehicles, as they exhibit strong tumor affinity and some antitumor immune activation. Treatment with TCVs loaded BAM15 effectively hampered tumor progression with promoting T-cell infiltration (Fig. 6). The broad influence of BAM15 on cellular metabolism suggests its capability to induce metabolic reprogramming across various tumors, highlighting the potential of BAM15-induced metabolic immune activation in advancing cancer therapy.

## Results

### Low-dose BAM15 induces futile energy expenditure in vitro

Initially, we evaluated the impact of BAM15 at various concentrations (0, 5, 50, 500, 5 K, and 50 K ng/mL) on the mitochondrial respiration of tumor cells. After 24 h of treatment, the 5 and 50 ng/mL doses of BAM15 increased both basal respiration and oxygen consumption related to proton leakage in tumor cells (Figs. 1A–C and S1A). Dosage at 500 ng/mL did not increase the basal respiratory oxygen consumption as expected, which may be due to 500 ng/mL is close to the IC50 of BAM15 on cells, and long-term treatment at 500 ng/mL BAM15 induces severe metabolic stress [23]. Dehydrogenase activity, an indicator of cell metabolic activity, was assessed via WST-8 kits [26]. The results indicated that 50 ng/mL BAM15 significantly enhanced dehydrogenase activity in 4T1 (Fig. 1D) and B16 (Fig. S1B) tumor cells without affecting cell growth (Figs. 1E, F and S1C, D). Thus, 50 ng/mL BAM15 was selected as the concentration for subsequent experiments. Our subsequent data revealed that 50 ng/mL BAM15 reduced ATP production (Figs. 1G and S1E) and the mitochondrial membrane potential (MMP) of tumor cells (Figs. 1H, I and S1F). Furthermore, we examined the activity of essential proteins associated with metabolism. The findings revealed that treatment with BAM15 for 24 h notably activated the pyruvate dehydrogenase complex (PDHa), along with modest increases in AKT and AMPK activity (Fig. 1J, K and S1G, H). These results suggest that BAM15 accelerates whole-cell energy utilization by driving mitochondrial oxidative metabolism. The prolonged rise in oxidative metabolism due to 50 ng/mL BAM15 did not lead to mitochondrial damage (Fig. 1L, 10% FBS group), possibly resulting from the compensatory enhancement of certain reductive metabolic pathways. By mimicking a nutrient-deficient tumor microenvironment through serum deprivation, and found that 50 ng/mL BAM15 treatment could induce tumor cell mitophagy (Fig. 1L, 1%FBS group, Fig. S1I), suggesting that low doses of BAM15 might also induce cellular stress in energy-deficient tumor tissues. Collectively, these results imply that treatment with 50 ng/mL BAM15 boosts catabolic processes while diminishing ATP production in tumor cells, resulting in an increase in unnecessary energy expenditure, as illustrated in the schematic diagram (Fig. 1M).

### BAM15-altered tumor secretions enhance T cell responses

The tumor immune microenvironment (TME) plays a crucial role in tumor development and is significantly influenced by metabolic enzymes and metabolites. To assess the effect of BAM15-modified tumor metabolism on immune function, tumor cells were treated with or without BAM15 for 12 h, after which the culture supernatants were collected. T cells and macrophages were isolated from tumor-bearing mice and exposed to the culture supernatants from DMSO-treated tumor cells (TC group) or BAM15-treated tumor cells (TC-BAM15 group). Immune cells treated with DMSO served as a control group, while the effects of BAM15 directly on immune cells were also evaluated (BAM15 group) (Figs. 2A and S2A, B). QPCR analysis of treated T cells indicated that the tumor cell supernatant (TC group) elevated the expression of LAG, Tigit, and PD-1 genes, with these increases being mitigated in the TC-BAM15 group (Fig. 2B), suggesting that tumor cell secretions contribute to T cell exhaustion, which BAM15 treatment alleviated by altering these secretions. Flow cytometry further revealed a higher proportion of CD8^+^ T cells in the TC-BAM15 group compared to the TC group (Fig. 2C, D). Additionally, Western blotting results demonstrated that T cells in the TC-BAM15 group exhibited elevated Granzyme B protein levels compared to the TC group (Fig. 2E, F), indicating enhanced T-cell cytotoxicity. Overall, BAM15 treatment augmented T-cell-mediated tumor destruction.

As shown in Fig. S2C, D, treatment with 50 ng/mL did not lead to macrophage death. Treatment with tumor secretions (TC group) altered several classical macrophage receptors/factors, including CD206, CD80, and CD163, in bone marrow-derived macrophages (macrophages φ, control group), and BAM15 treatment did not reverse tumor-induced macrophage differentiation (TC-BAM15 group) (Fig. S2E). Given the complex roles of tumor-associated macrophages and the limited number of genes analyzed, our current assessment does not demonstrate that BAM15 preconditioning enhances the anti-tumor activity of macrophages (Fig. S2E).

### Intratumoral administration of BAM15 enhances oxidative metabolism in vivo

Next, we established a bilateral tumor-bearing mouse model to evaluate the impact of BAM15 on tumor metabolism in vivo. BAM15 at a concentration of 50 ng/mL was injected intratumorally on the right side, followed by a tail vein injection of ^18^F-FDG 10 min later. Glucose uptake by the bilateral tumors was monitored via PET/CT 20 min post-injection (Fig. S3A). The results demonstrated that intratumoral BAM15 injection significantly elevated FDG uptake in the right tumor (Fig. 3A–D, and S3B, C). Subsequent infrared thermal imaging showed an increase in thermal radiation in the tumors injected with BAM15 (Fig. 3E, F), indicating that BAM15 treatment enhances the conversion of chemical energy into heat. Due to the imaging limitations in C57BL/6 mice, which have black skin, we chose to use BALB/c mice bearing 4T1 tumors for the subsequent experiments. Photoacoustic computed tomography (PACT) was employed to measure blood oxygen saturation (sO_2_), revealing lower sO_2_ in larger tumors (mouse 3 > mouse 2 > mouse 1) (Fig. 3G). Following BAM15 injection, sO_2_ concentrations were reduced in tumors of all sizes (Figs. 3G and S3D). Furthermore, the activity of key proteins involved in metabolism was assessed, and BAM15 was found to significantly activate the pyruvate dehydrogenase complex (PDHa), accompanied by slight increases in AKT and AMPK activity (Fig. 3G–F). These changes, along with increased glucose uptake, oxygen consumption, thermal radiation, and activation of metabolic pathways, suggest a rapid acceleration in oxidative metabolism.

### BAM15 treatment promotes catabolism in tumor tissue

Metabolomics analysis was used to evaluate the metabolic effects of BAM15 treatment in vivo. Principal component analysis (PCA) revealed a distinct metabolic profile post-BAM15 treatment (Fig. 4A). KEGG pathway enrichment analysis indicated significant upregulation of the pentose phosphate pathway (PPP), glycolysis pathway, and carbon metabolism pathway (Fig. 4B), all pointing to enhanced catabolic activity. Conversely, the downregulation of central carbon metabolism (CCM) and amino acid biosynthesis pathways suggests a suppression of anabolic processes (Fig. 4B). Differentially abundant metabolite analysis showed a substantial increase in glucose uptake (Fig. 4C, D), while glutamine concentration remained relatively unchanged (Fig. 4E). Quantitative PCR (qPCR) analysis revealed that BAM15 treatment upregulated the transcription of *hexokinase 1/2* (Figs. 4F, G) and 6-*phosphogluconate dehydrogenase (6PGD)* (Fig. 4H), while downregulating the fatty acid transport gene *FABP5* (Fig. 4I) and *lactate dehydrogenase* (LDH) levels (Fig. 4J). These results confirm that BAM15 treatment shifts tumor metabolism towards increased glucose utilization. Subsequent experiments demonstrated that BAM15 treatment led to elevated reactive oxygen species (ROS) production in tumor cells (Fig. 4K–L), indicating increased oxidative stress. Slight downregulation of the tricarboxylic acid (TCA) cycle (Fig. 4C, D) may be a consequence of mitochondrial damage from the ROS burst, while the upregulated PPP likely serves to protect cells from excessive oxidative stress by producing NADPH [27, 28] (Fig. 4M).

### BAM15 enhances T cell cytotoxicity partly dependent on mitochondrial metabolites accumulation

BAM15 treatment altered several mitochondrial enzyme-dependent metabolites (Fig. 5A). Notably, the most significant increase in glutarylcarnitine (C5DC) production, a marker of severe mitochondrial metabolic dysfunction, was observed in the blocked tryptophan and lysine degradation pathway (Catalyzed by mitochondrial glutaryl-CoA dehydrogenase) (Fig. 5B). Metabolomic analysis further revealed an accumulation of substrates cleaved by mitochondrial hydrolases, while products catalyzed by mitochondrial enzymes were significantly reduced in the BAM15-treated group. These abnormalities were predominantly observed in amino acid (Fig. 5C) and lipid metabolism (Fig. 5D). Electron microscopy (Fig. 5E) confirmed the occurrence of severe structural damage under BAM15 treatment. Additionally, levels of the final products of fatty acid oxidation, 3-methyladipic acid and heptanedioic acid, were significantly decreased (Fig. 5F). The accumulation of linoleic acid (α-LA) (Fig. 5D, G) was possibly due to the inhibition of β-oxidation, which reduces its specific metabolites, such as hydroxyoctadecadienoic acid (HOA) [29] (Fig. 5H). The downregulation of fatty acid utilization further indicates that BAM15 treatment impairs the metabolic flexibility of tumors (Fig. 4). Notably, α-LA has been shown to inhibit T cell exhaustion in tumor-infiltrating effector memory T cells [30]. Immunoblotting of isolated T cells revealed that α-LA directly promotes the expression of granzyme B (Fig. 5I), and the increased granzyme B expression in treated tumor tissues may, in part, be attributed to the accumulation of α-LA (Fig. 5J). Thus, BAM15 not only induces metabolic stress but also enhances T cell-mediated tumor killing.

### BAM15 delivered by TCV can effectively elicit robust T cell response and inhibit tumor growth

We demonstrated that local injection of BAM15 impedes multi-source nutrient utilization and enhances T cell cytotoxicity. To extend this therapeutic approach to systemic tumor treatment without causing whole-body weight loss, we engineered T lymphocyte-derived cellular vesicles (TCVs) to deliver BAM15 to tumors. Through ultrasonic crushing and nano-extruder extrusion, tumor-infiltrating T lymphocytes were transformed into nanovesicles with a uniform particle size, typically ranging from 100 to 200 nm, as confirmed by transmission electron microscopy (TEM) (Fig. S4A) and nanoparticle tracking analysis (NTA) (Fig. S4B). This size range is deemed favorable for effective tumor enrichment and retention. Surface potential, a critical parameter influencing nanoparticle stability and longevity, was evaluated and determined to be -16 mV for TCVs (Fig. S4C). This ensures their stability in a PBS buffer solution for over 7 days (Fig. S4D). To evaluate the PD-1 content in TCVs, nanoflow analysis revealed that 88.6% of tumor-bearing mouse TCVs were PD-1 positive (Fig. S4E), indicating promising tumor-targeted drug delivery potential.

To validate the tumor selectivity of TCVs, DiO-labeled TCVs were tested for binding and internalization. TCVs efficiently bound to tumor cell surfaces (Fig. S4F) and were progressively internalized over time (Fig. S4G). In comparison to vascular endothelial cells, TCVs exhibited higher internalization efficiency in tumor cells (Fig. S4H, I), confirming their tumor selectivity at the cellular level.

To assess the in vivo targeting ability of TCVs, ICG-labeled TCVs were injected into the tail vein of tumor-bearing mice. Fluorescence imaging of major organs and tumor tissues 24 h post-injection showed significantly higher fluorescence intensity in tumors from the TCV-ICG group compared to the free ICG group (Fig. S4J). Longitudinal imaging of TCV distribution revealed that free ICG was metabolized within 9 h, while TCVs loaded with ICG persisted longer and accumulated in the tumor (Fig. S4K). These results confirm the ability of TCVs to effectively target and remain in the tumor microenvironment.

Like 50 ng/mL BAM15, 76 ng/mL TCV-BAM15 (about 66% loading efficiency and calculated as BAM15 concentration before packaging) treatment reduces mitochondrial potential by about 60% in 4T1 tumor cells (Fig. S5A, B). To evaluate its therapeutic potential, subcutaneous tumors were established in mice, and TCV-BAM15 was administered intravenously every 3 days for 5 consecutive sessions. Tumor size was monitored over time (Fig. 6A), and the tumor growth curve indicated that both TCV and TCV-BAM15 could inhibit tumor growth, with TCV-BAM15 demonstrating a more pronounced effect. (Fig. 6B, C). Notably, the inhibitory effect of TCV-BAM15 on tumor growth was visually evident in ex vivo tumor photographs (Fig. 6D). Ex vivo tumor weight further substantiated the inhibitory impact of TCV-BAM15 (Fig. 6E). Interestingly, BAM15 alone did not exhibit significant antitumor effects, likely due to rapid metabolism and poor tumor penetration. Despite a marginal weight loss caused by TCV-BAM15 (Fig. 6F), no significant difference in net body weight was detected compared with that in the control group (Fig. 6G). Furthermore, even though TCV loading might extend the body cycle (Fig. S4K) and prolong the liver/kidney/tumor residence time (Fig. S4J) of BAM15, no liver (Fig. S5C) or other tissue toxicity (Fig. S5D) was detected, confirming its safety profile. Investigate the antitumor mechanism, western blot analysis was performed on tumor tissues to assess the expression of cleaved caspase-3, PD-1, and granzyme B. Both TCV and TCV-BAM15 treatments induced the expression of caspase-3 and granzyme B, with TCV-BAM15 showing the most robust effect. Immunofluorescence analysis of tumor tissues demonstrated that both TCVs and TCV-BAM15 increased T-cell infiltration (Fig. 6J). The elevated granzyme B expression and T-cell infiltration indicate an activated immune response and tumor cell cotoxic. TUNEL and Ki67 staining further revealed varying degrees of tumor cell death induced by TCV and TCV-BAM15 (Fig. 6J). Taken together, TCV-BAM15 mitigates the immunosuppressive tumor microenvironment by impeding tumor metabolism, thereby enhancing the immune cell response (Fig. 6K).

## Discussion

Starvation-based therapies, which rely on energy restriction, have become a popular approach in cancer treatment. The fasting-mimicking diet has shown promise as an adjunctive therapy to enhance the effectiveness of chemotherapy, radiotherapy, and immunotherapy in preclinical models [31, 32]. Clinical trials have demonstrated that antiangiogenic drugs, such as bevacizumab [33] and cabozantinib [34], can improve survival in cancer patients when used in combination with conventional chemotherapy. However, their efficacy as monotherapies in patients with solid tumors has been limited. Metabolic inhibitors like IMT1 B [35], Mito-LND [36], and nebivolol [37] have not yet been successfully identified or utilized in clinical settings [38]. These findings underscore that many existing starvation-based therapies are insufficient for achieving effective nutritional restriction in tumors.

By uncoupling mitochondrial function with BAM15, the potential energy stored in the inner mitochondrial membrane was expended uncontrollably, leading to the compensatory activation of key productive enzymes in tumor cells. Infrared thermal imaging revealed that BAM15 treatment elevated the temperature of tumor tissue, attributed to the conversion of mitochondrial potential energy into heat. As a result, tumor energy depletion was intensified, even as BAM15 increased glucose uptake. The associated increase in mitochondrial damage and ROS further exacerbated metabolic stress, which was consistent with the upregulation of LC3BII/I. While our imaging data revealed acute hypoxia upon BAM15 administration, whether this effect persists chronically remains unclear due to experimental limitations. However, prior studies suggest that mitochondrial uncouplers may eventually alleviate hypoxia by p53 pathways [39]. Moreover, anabolic pathways were suppressed due to energy limitation, while the pentose phosphate pathway was enhanced to mitigate oxidative stress. The metabolic regulatory function of BAM15 was validated in common tumor models, such as 4T1 and B16 tumors, further supporting the universality of BAM15’s metabolic regulation and its ability to overcome differential responses in various tumor types.

The antitumor effects of CD8^+^ T cells may be partly attributed to the elevated levels of LA, which have been reported to prevent T-cell exhaustion and enhance antitumor T-cell responses [30]. The decrease in 3-hydroxyanthranilic acid [40] and other altered metabolites may also contribute to the antitumor potency of effector T cells. Further investigation of T-cell activators among these differentially abundant metabolites holds great promise for enhancing immune therapy.

Immune cells are vital in defending against tumor progression, and tumor cells often create immunotherapy-resistant reservoirs to manipulate immune cells in the tumor microenvironment. Local hypoxia, induced by cancer cells, exacerbates T-cell exhaustion and impairs T-cell cytotoxicity [41]. while metabolites from the TCA cycle influence the T/Treg ratio [12, 13]. The metabolic reprogramming triggered by BAM15 leads to new metabolic secretions from tumor cells that enhance T-cell effector functions by preventing exhaustion. This results in an improved ratio of CD8^+^ T cells and increased granzyme B expression. The antitumor effects of CD8^+^ T cells may be partly attributed to the elevated levels of LA, which have been reported to prevent T-cell exhaustion and enhance antitumor T-cell responses [30]. The decrease in 3-hydroxyanthranilic acid [40] and other altered metabolites may also contribute to the antitumor potency of effector T cells. Further investigation of T-cell activators among these differentially abundant metabolites holds great promise for enhancing immune therapy.

BAM15-induced metabolic reprogramming reshapes the tumor microenvironment (TME), shifting it from cooperative to competitive modes of cellular interaction. However, the clinical application of BAM15 in antitumor therapy faces substantial challenges due to its rapid clearance and suboptimal tumor-targeting efficiency. Biological vesicles have been extensively studied as drug delivery vectors for tumor therapy [42]. By utilizing adhesive molecules and chemokine receptors on their surface, biomimetic nanovesicles can specifically target inflamed vasculature, enhancing the accumulation of therapeutic agents in tumors [43–45]. Unlike conventional pharmacological approaches, cell-based therapies employing biomimetic nanovesicles offer a promising strategy to reduce toxicity and improve drug delivery efficiency [46]. Lymphocyte-derived microparticles (LMPs) have emerged as effective biomimetic nanovesicles for therapy, exhibiting both antiangiogenic properties and the ability to induce cell cycle arrest. Here, T cell vesicles (TCVs) loaded with indocyanine green (ICG) were shown to have high tumor affinity and long-term tumor retention, characterizing them as an effective nanoparticle platform. Intravenous administration of BAM15-loaded TCVs (TCV-BAM15) significantly inhibited tumor growth. Leveraging the unique properties of TCVs, BAM15-mediated metabolic modulation avoids the physiological risks associated with non-specific metabolic interventions or traditional metabolic inhibition drugs, thereby broadening its potential for multi-tumor therapy.

## Materials and methods

### Materials

BAM15 was purchased from Aladdin Biochemical Technology (B287299, Shanghai). FITC-conjugated anti-CD80, PE-conjugated anti-CD206, and FITC-conjugated anti-CD206: obtained from 4A Biotech Co., Ltd. PE-conjugated anti-iNOS: Thermo Fisher Scientific, Inc. phospho-PDHa1/2 (Ser293/291), phospho-AMPKa (Thr172), phospho-AKT1/2/3 (Ser473), tubulin, and actin antibodies were purchased from Affinity Bioscience. LC3B was from Hangzhou Huaan Biotechnology Co., Ltd. HRP-conjugated AffiniPure goat anti-rabbit IgG (SA00001-2) and HRP-conjugated AffiniPure goat anti-mouse IgG (SA00001-1) were obtained from Proteintech Group, Inc. Annexin V-FITC/PI Apoptosis Detection Kit and WST-8 Kit were buy from Dojindo Laboratories. ATP Assay Kit was obtained from Beyotime (S0026) and used according to the manufacturer’s protocol.

### Animal models

All animal studies were approved by the Institutional Animal Care and Use Committee of the Guangdong Medical Laboratory Animal Center (KY2023-039-01). Four to six-week-old BALB/c and C57BL/6N mice were purchased from Guangdong Medical Laboratory Animal Center. For the tumor-bearing model, 2 × 10^6^ cells suspended in 200 μL of normal saline were subcutaneously implanted into mice.

Bilateral tumor-bearing mice were used to visualize the metabolic regulatory effects of BAM15. Once the tumor reached ~4 × 4 mm in size, a single intratumoral injection of 10 μL of BAM15 (50 ng/mL) was administered into the right tumor, while 10 μL of solvent was injected into the left tumor. PET/CT imaging, infrared thermal testing, and photoacoustic tomography (PACT) were performed at appropriate time points.

For tumor metabolomics, 10 μL of BAM15 (50 ng/mL) was injected intratumorally daily for three consecutive days. After euthanizing the mice, tumor tissues were excised and immediately frozen in liquid nitrogen. The HM Pro 2300 Metabolomics Project aims at 700 small metabolites and more than 1600 lipids were performed by BGI Tech Solutions Co., Ltd.

To investigate the antitumor efficacy of BAM15 in vivo, the mice were randomly divided into four groups: control, BAM15, T lymphocyte-derived cellular vesicle (TCV), and TCV-BAM15 groups. Once tumors size reached ~4 × 4 mm, the mice were intravenously injected with 100 µL of normal saline (control group), 7.5 mg/kg BAM15 (7.5 = 0.05 μg/mL (the concentration of cell processing) × 5 mL/kg (constant fluid uptake in mice)/0.05 kg (body weight of mice)) (BAM15 group), 100 μL of T lymphocyte-derived cellular vesicles (TCV group) or 11.5 mg/kg TCV-BAM15 (about 66% loading efficiency, TCV-BAM15 group) once every 3 days. The tumor size was monitored before each injection. The tumor size was evaluated according to the calculation formula *V* = (*L* × *W*^2^)/2, where L is the length (longest dimension) and W is the width (shortest dimension). Tumor growth curves were plotted within 18 days, when the first lethal-sized tumor was detected in the control group.

### Assessment of mitochondrial respiration

The XF Cell Mito Stress Test Kit (Agilent) was used to evaluate the effects of BAM15 on mitochondrial respiration. 4T1 or B16 cells were seeded at a density of 1500 cells per well in an XFe 96-well plate (Agilent) and cultured for 24 h in DMEM or RPMI-1640 complete medium (10% FBS and 1% penicillin-streptomycin). The medium was then replaced with fresh complete medium containing various concentrations of BAM15 (0, 5, 50, 500, 5000, or 50,000 ng/mL). After 24 h, the cells were washed and incubated for 1 h in BAM15-containing XF basal medium (pH 7.4) supplemented with 1 mM pyruvate, 2 mM glutamine, and 10 mM glucose at 37 °C without CO_2_. The cells were subsequently treated with serial injections of oligomycin (1.5 μM), FCCP (0.5 μM), and rotenone (0.5 μM). Mitochondrial respiration parameters were calculated according to the XF Cell Mito Stress Test Kit protocol.

### Detection of cell dehydrogenase activity

Dehydrogenase activity was evaluated using a WST-8 assay, which measures the reduction of WST-8 to formazan. The absorbance at 450 nm is directly proportional to the activity of dehydrogenases. Briefly, 4T1 or B16 cells were seeded in a 96-well plate at a density of 1500 cells per well and cultured for 24 h. The cells were then treated with BAM15 at various concentrations. After 6 h, the medium was replaced with fresh complete medium supplemented with WST-8 and BAM15. The cells were incubated at 37 °C in a 5% CO_2_ atmosphere for 1 h, and the absorbance at 450 nm was measured using a Spark Multimode Reader (TECAN, Switzerland).

### Cell cycle analysis

Cells were seeded in 6-well plates and treated with 50 ng/mL BAM15 for 24 h. After treatment, the cells were fixed with 70% ethanol and pretreated with 250 μg/mL RNase. Propidium iodide (PI, 50 μg/mL) staining was used to quantify cell proliferation. Flow cytometry analysis was performed using a FACS Canto flow cytometer (Becton Dickinson, Mountain View, CA) with excitation at 488 nm. The cell cycle profile was determined using the FlowJo software.

### Mitochondrial potential staining

JC-1 was used to monitor the mitochondrial potential because its color changes in a manner dependent on the magnitude of the membrane potential. Briefly, cancer cells were seeded on 6-well plates or confocal dishes for 24 h and then incubated with DNP, BAM15, or insulin for another 6 h. JC-1 was purchased from Thermo Fisher (T3168), and staining was performed according to the manufacturer’s protocol. FACS or 3D fluorescence microscopy was used to observe the JC-1 signal.

### Isolation and culture of tumor-infiltrating lymphocytes

4T1 cells were inoculated subcutaneously on the backs of mice. Tumors were excised under sterile conditions once they reached ~1 cm in diameter. The tumors were cut into small fragments using sterile scissors and digested with cathepsin for 5–10 min to disaggregate the tissue. The digested tissue was processed to achieve a single-cell suspension, which was filtered through a 200-mesh screen. The resulting cell suspension was centrifuged at 1500 rpm to collect the cell pellet. CD8^+^ T cells were isolated using a CD8^+^ T-cell sorting kit according to the manufacturer’s protocol. The isolated T cells were cultured and expanded in RPMI 1640 medium supplemented with 200 U/mL IL-2.

### Quantitative PCR (qPCR)

Total RNA from tissues and cells was extracted using TRIzol Reagent (R401-01, Vazyme) and reverse transcribed into cDNA using a high-capacity cDNA reverse transcription kit (Promega, United States). Gene expression levels were quantified using real-time fluorescence quantitative polymerase chain reaction (qPCR) on a LightCycler® 480 Instrument II (Roche, Sweden) with SYBR Green Master Mix (Promega, United States). The primer sequences used for quantitative RT-qPCR are listed in Supplementary Table 1.

### Western blot analysis

Western blot analyzes were conducted to measure the levels of key proteins. Total protein from cells was extracted using RIPA buffer, while tissue protein was prepared by additional chopping with a sample freezing grinder (LUKYM-I) for 90 s at 70 Hz. Equal amounts of protein were separated on 10% SDS-polyacrylamide gels and transferred to PVDF membranes. The membranes were incubated overnight at 4 °C with specific primary antibodies, followed by a 1-h incubation with HRP-conjugated secondary antibodies at room temperature. Protein signals were visualized and analyzed using a ChemiDoc Imaging System (Bio-Rad).

### PET/CT

Mice were fasted overnight before the procedure, then weighed, and anesthetized via intraperitoneal injection of pentobarbital. BAM15 or an equal volume of solvent was intratumorally injected into the mice. Ten minutes later, ^18^F-FDG (X5 μCi per gram of body weight) was administered via tail vein injection. After 20 min, a 30-min static PET scan (Siemens/Concorde Medical Solutions, New York, NY, USA) was performed, followed by a 10-min CT scan (MicroCAT II, CTI Siemens, Munich, Germany), conducted by a professional operator.

### Photoacoustic computed tomography (PACT)

A PACT system (SIP-PACT, Union Photoacoustic Technologies) was used for organ cross-sectional imaging. Detailed parameters of the system have been described previously [47, 48]. The system’s high-frequency scanning speed (20 Hz) and multiwavelength coupling structure (680–950 nm, 1064 nm, and 1190–2600 nm) allowed accurate measurement of sO_2_ levels. During the procedure, each mouse was anesthetized with 2% isoflurane and positioned upright in the center of the imaging tank. Images at 1064 nm were captured for positioning, while wavelengths of 750 nm and 850 nm were used to calculate sO_2_ levels.

### Temperature measurements

Skin temperature around the tumor was measured using an infrared thermal imaging camera (E60: Compact Infrared Thermal Imaging Camera, FLIR). Data were analyzed with FLIR ResearchIR Max 3.4 software (FLIR, West Malling).

### Preparation and characterization of TCVs

The preparation of TCVs was adapted with slight modifications from previously reported methods. Briefly, 10^7^ T lymphocytes infiltrated with tumors were lysed using liquid nitrogen. The cells were then disrupted into fragments via ultrasonication and extruded into nanovesicles using a liposome extruder, resulting in particles ~200 nm in diameter. To enhance the loading efficiency of BAM15, it was added prior to ultrasonication at a ratio of 2 mg of BAM15 per 10^7 ^T cells. Mass spectrometry measured the BAM15 loading efficiency to be ~66%. The nanovesicles were separated via ultracentrifugation at 100,000 × *g* for 90 min. The resulting pellet was resuspended in PBS for further use.

For morphological analysis, TCVs were diluted in PBS, applied to a copper grid, and allowed to stand for 1 min before excess liquid was removed. The samples were negatively stained with 2% phosphotungstic acid for 10 s, and the morphology of the vesicles was observed using transmission electron microscopy (TEM). The particle size distribution and surface potential of the TCVs were characterized via nanoparticle tracking analysis (NTA, ZS90, Malvern) and zeta potential measurement (Litesizer DLS 500, Anton Paar). The presence of PD1 on the surface of TCVs was detected via nanoflow cytometry.

### Tumor cell affinity and internalization of TCVs

To assess TCV internalization, live tumor cells were incubated with TCVs for specified durations, after which uninternalized TCVs were washed away. The internalization was then visualized via confocal microscopy following staining with ginkin cyclic peptide and DAPI. Comparative analysis of TCV uptake by tumor cells (4T1) and normal cells (vascular endothelial cells, HUVECs) was performed by incubating both cell types with TCVs for identical durations. Fluorescence intensity was measured using flow cytometry to evaluate relative uptake levels.

### Statistical analysis

In vivo experiments were conducted with 5–7 mice randomly assigned to each group to minimize individual variability. For in vitro experiments, a minimum of three biological replicates were performed, with n representing independent replicates. Statistical analyzes were carried out using GraphPad Prism (version 9.0), and data are presented as the mean ± SEM. Two-group comparisons were analyzed using unpaired two-tailed Student’s *t*-tests (Figs. 1–6 and S1–5). One-way ANOVA with Tukey’s post hoc test was used for multiple comparisons involving two or more independent variables (Fig. 6). Statistical significance was denoted as follows: ***P < 0.001, **P < 0.01, and *P < 0.05 were considered statistically significant vs the control group. ##*P* < 0.01, #*P* < 0.05 were considered to indicate statistical significance vs the TCV group.

## Supplementary information

Supplemental material

## Data Availability

The datasets generated and/or analyzed during this study are included in the manuscript (and its supplementary files). Additional data are available from the corresponding author upon reasonable request.
