# Supplementary material for: Tumor metabolome remolded by low dose mitochondrial uncoupler elicites robust CD8+ T cell response
Source: Cell Death Discov. 2025 Jul 1;11:291. doi: 10.1038/s41420-025-02584-9 (PMC12215621; doi:10.1038/s41420-025-02584-9)
Supplement: Supplementary file 1 — Supplemental material [file 41420_2025_2584_MOESM1_ESM.pdf]

Supplementary information

Tumor Metabolome Remolded by Low Dose Mitochondrial Uncoupler Elicites Robust $CD8^+$ T Cell Response

Xiaoxiao Jiang[1] †, Zhijin Fan[1,2] †, Zhenzhen Zhang[3], Fanchu Zeng[1], Tong Sun[1], Yuchen Li[3], Guojia Huang[1] and Liming Nie[1,2] *

1. Medical Research Institute, Guangdong Provincial People's Hospital (Guangdong Academy of Medical Sciences), Southern Medical University, Guangzhou, 510080, China.

2. School of Medicine, South China University of Technology, Guangzhou, 510006, China.

3. Key Laboratory of Brain, Cognition and Education Sciences, Ministry of Education, China; Institute for Brain Research and Rehabilitation, and Guangdong Key Laboratory of Mental Health and Cognitive Science, South China Normal University, Guangzhou, China.

*Corresponding Authors: nieliming@gdph.org.cn.

†These authors contributed equally to this work.

Figure legends:

Figure s1. Low dose of BAM15 increased futile energy expenditure in B16 tumor cell. A. Oxygen consumption rates of B16 tumor cells after 16 hours exposure at varying concentrations (0, 5, 50, 500, 5K, 50k ng/mL) of BAM15 (N=7). **B.** Changes of Basal oxygen consumption rates under described dasages of BAM15. **C.** Changes of proton leak oxygen consumption rates under described dasages of BAM15. **D.** Mitochondrial dehydrogenase activity detection by using WST-8 at varying dosages of BAM15 (n=6). **E.** Cell cycle analysis with or without 50 ng/mL BAM15 (n=3). **F.** Quantitation of each phase by PI staining. **G.** ATP concentration measurement under DMSO, Insulin, BAM15, DNP treatment in B16 cell (n=5). **H.** Mitochondrial potential staining of B16 cell under BAM15, DNP, or Insulin treatment. **I.** Phosphoration of AMPK, AKT,and PDHa were measuresd by westernbloting. **J.** Quantitative of the ratio of phospho-protein/ GAPDH showed in I. Data are normalized to control group and expressed as mean ± SEM, $n \geq 3$. ***P <0.001, **P < 0.01, and *P < 0.05 were considered to be statistically significant vs Control group.

Figure s2. A. Flow cytometry was used to analyze the proportion of macrophages φ in isolated bone marrow-derived cells after 7 days stimulation with M-CSF. **B.** Schematic of the experimental design. **C.** FACS analysis and **D.** apoptosis caculation of macrophages φ with or without 50 ng/mL BAM15 incubation for 12 hours. **E.** Gene expression of *CD80, CD206, CD163, IL-4, Siflec1* and *Pcsk9* in macrophages treated with or without 50 ng/mL BAM15 incubation by QPCR. Data are normalized to control group and expressed as mean ± SEM, NS was considered to be non-significant.

Figure s3. A. Schematic of the experimental design. **B.** Sagittal-section of tumors with or without BAM15 intratumaly injection by PET/CT imaging for 4T1 or **C.** B16 tumors. **D.** Relative decrease of sO2 by BAM15 injection.

Figure s4. Characterization of T lymphocyte-derived cellular vesicles (TCVs). A. Transmission electron microscopy imaging of the TCV. **B.** Particle size distribution of the TCV. **C.** Surface potential of TCVs. **D.** Stability of TCVs in PBS. **E.** The proportion of PD1 positive vesicles was detected by nanoflow analysis. **F.** Affinity of TCVs for tumor cells. **G.** Tumor cell-internalized TCVs were observed via fluorescence imaging. **H.** Flow cytometry was used to compare the efficiency of TCE uptake by tumor cells and vascular endothelial cells. **I.** The distribution of TCVs in major organs 24 h after caudal vein injection was detected via fluorescence imaging. **J.** Ex vivo fluorescence imaging of heart liver spleen and lung kidney tumors. **K.** The tumor enrichment process of TCVs was monitored by fluorescence imaging in vivo.

Figure s5. A. TCV-BAM15 treatment decreased mitochondrial potential of 4T1 cell detected by FACS. **B.** Mitochondrial potential calculation of 4T1 cell with indicated treatment. **C.** TCV-BAM15 treatment did not affect liver and kidney function. **D.** Safety testing of TCV-BAM15 treatment. Group differences were assessed by two-tailed t-tests with data normalized to controls (mean ± SEM, n ≥ 3). Significance levels: **P < 0.01, *P < 0.05 vs control; ##P < 0.01 vs TC; NS was considered to be non-significant.

Figure s1

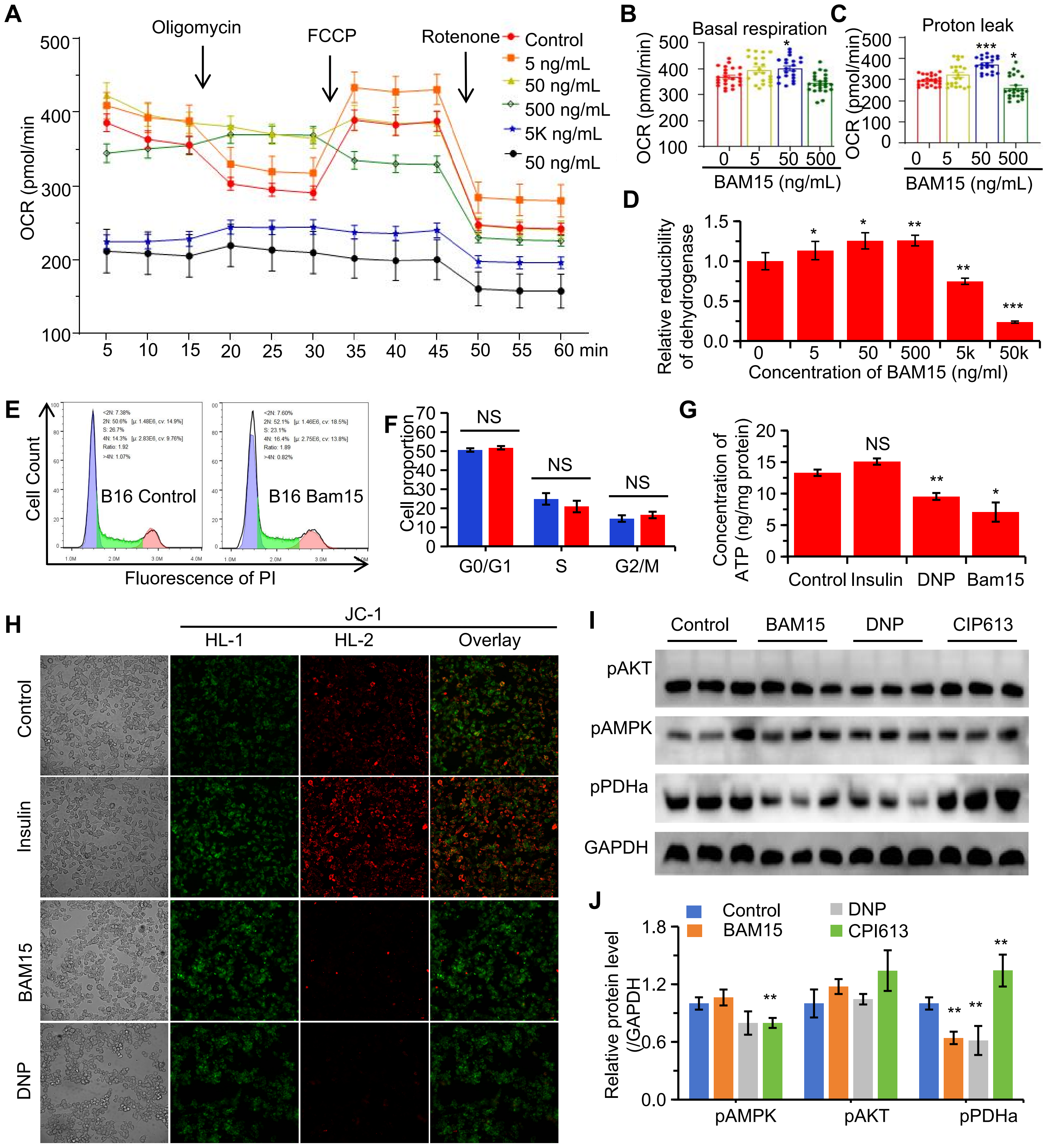

A
Oligomycin
FCCP
Rotenone
Control
5 ng/mL
50 ng/mL
500 ng/mL
5K ng/mL
50 ng/mL
OCR (pmol/min
60 min
B
Basal respiration
OCR (pmol/min)
BAM15 (ng/mL)
C
Proton leak
OCR (pmol/min)
BAM15 (ng/mL)
D
Relative reducibility of dehydrogenase
Concentration of BAM15 (ng/ml)
E
Cell Count
B16 Control
B16 Bam15
Fluorescence of PI
F
Cell proportion
NS
G0/G1
S
G2/M
G
Concentration of ATP (ng/mg protein)
NS
Control
Insulin
DNP
Bam15
H
JC-1
HL-1
HL-2
Overlay
Control
Insulin
BAM15
DNP
I
Control
BAM15
DNP
CIP613
pAKT
pAMPK
pPDHa
GAPDH
J
Control
BAM15
DNP
CPI613
Relative protein level (/GAPDH)
pAMPK
pAKT
pPDHa

Figure s2

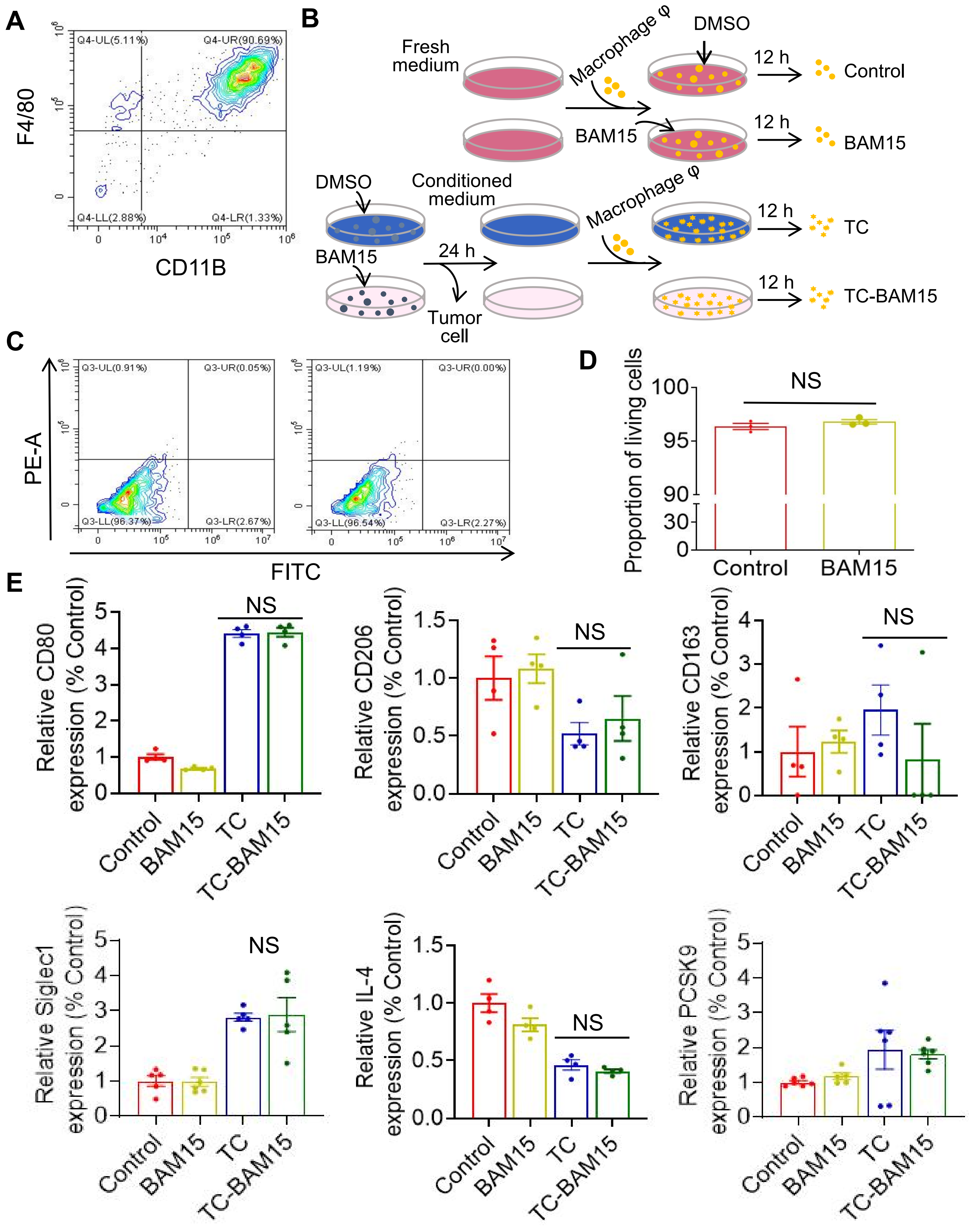
A
F4/80
CD11B
Q4-UL(5.11%)
Q4-UR(90.69%)
Q4-LL(2.88%)
Q4-LR(1.33%)
B
Fresh medium
Macrophage φ
DMSO
12 h
Control
BAM15
12 h
BAM15
DMSO
Conditioned medium
Macrophage φ
12 h
TC
BAM15
24 h
Tumor cell
12 h
TC-BAM15
C
PE-A
FITC
Q3-UL(0.91%)
Q3-UR(0.05%)
Q3-LL(96.37%)
Q3-LR(2.67%)
Q3-UL(1.19%)
Q3-UR(0.00%)
Q3-LL(96.54%)
Q3-LR(2.27%)
D
Proportion of living cells
NS
Control
BAM15
E
Relative CD80 expression (% Control)
Relative CD206 expression (% Control)
Relative CD163 expression (% Control)
Relative Siglec1 expression (% Control)
Relative IL-4 expression (% Control)
Relative PCSK9 expression (% Control)
NS
Control
BAM15
TC
TC-BAM15

Figure s3

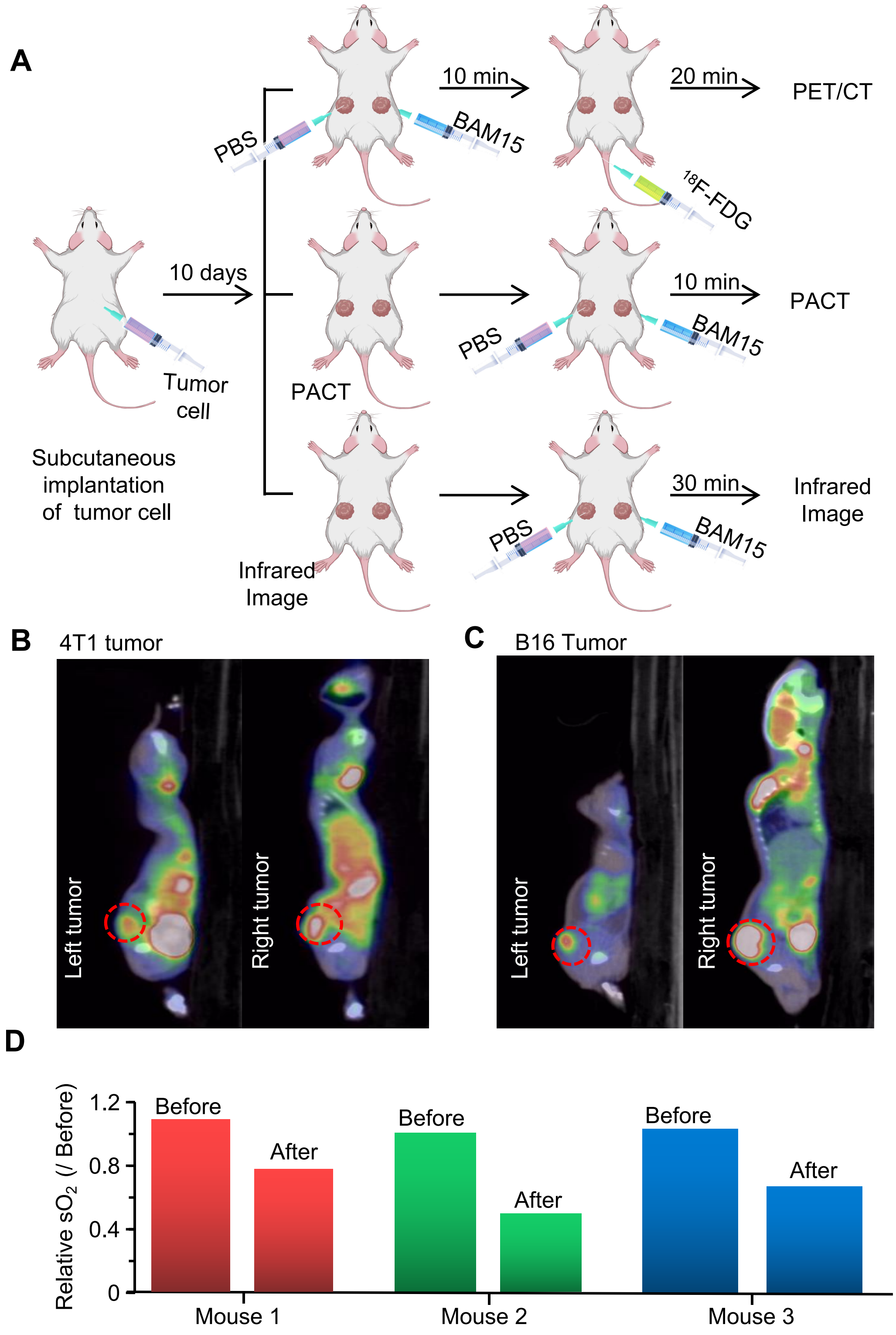
A
PBS
BAM15
10 min
20 min
PET/CT
18F-FDG
10 days
Tumor cell
Subcutaneous implantation of tumor cell
PACT
PBS
10 min
BAM15
PACT
Infrared Image
PBS
30 min
BAM15
Infrared Image
B
4T1 tumor
Left tumor
Right tumor
C
B16 Tumor
Left tumor
Right tumor
D
Relative sO2 (/ Before)
1.2
0.8
0.4
0
Before
After
Before
After
Before
After
Mouse 1
Mouse 2
Mouse 3

Figure S4

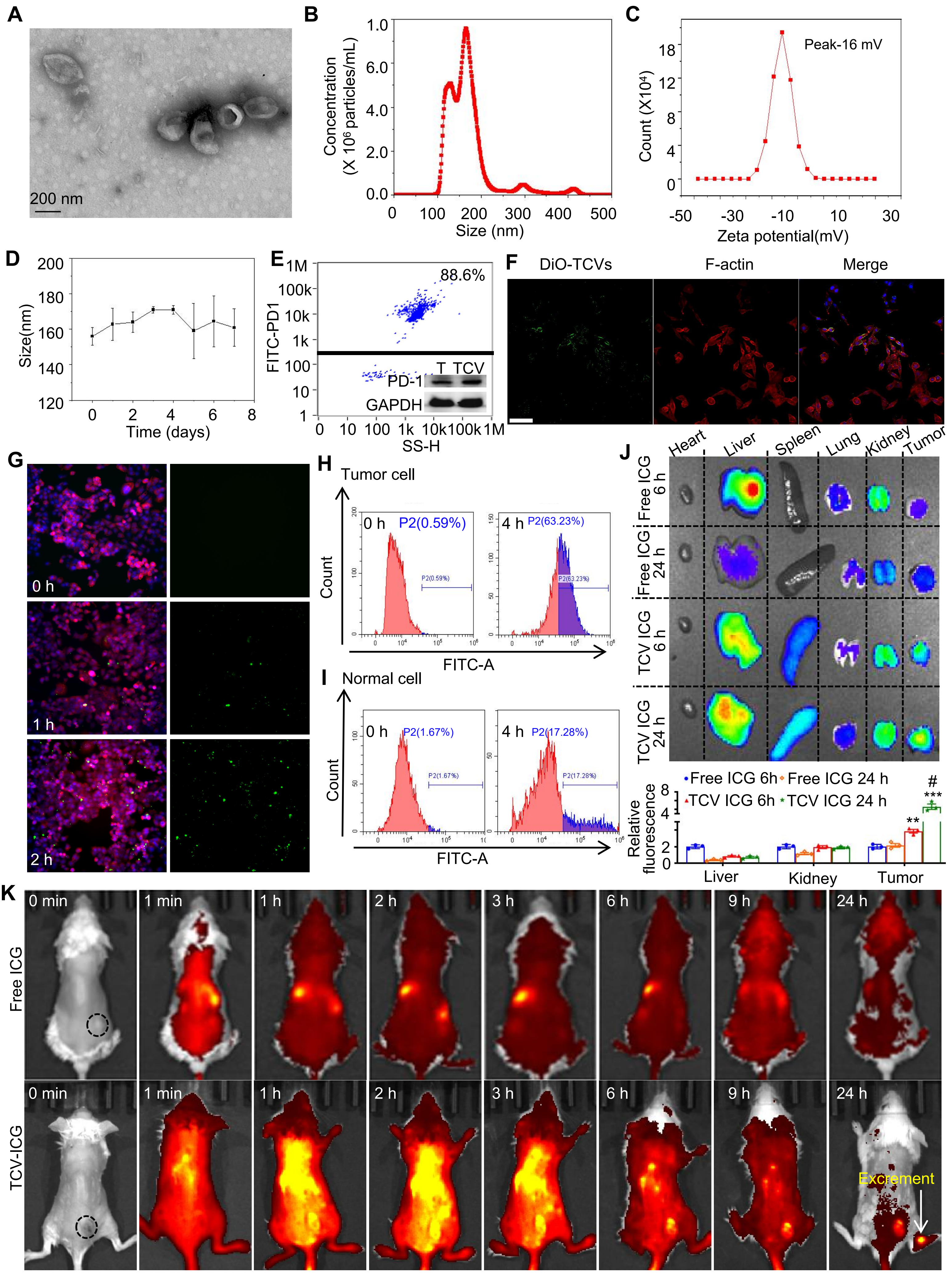

A
200 nm
B
Concentration (X 10^6 particles/mL)
Size (nm)
C
Count (X10^4)
Peak-16 mV
Zeta potential(mV)
D
Size(nm)
Time (days)
E
FITC-PD1
88.6%
SS-H
T TCV
PD-1
GAPDH
F
DiO-TCVs
F-actin
Merge
G
0 h
1 h
2 h
H
Tumor cell
0 h P2(0.59%)
4 h P2(63.23%)
Count
FITC-A
I
Normal cell
0 h P2(1.67%)
4 h P2(17.28%)
Count
FITC-A
J
Heart
Liver
Spleen
Lung
Kidney
Tumor
Free ICG 6 h
Free ICG 24 h
TCV ICG 6 h
TCV ICG 24 h
Free ICG 6h Free ICG 24 h
TCV ICG 6h TCV ICG 24 h
Relative fluorescence
Liver
Kidney
Tumor
K
Free ICG
TCV-ICG
0 min
1 min
1 h
2 h
3 h
6 h
9 h
24 h
Excrement

Figure s5

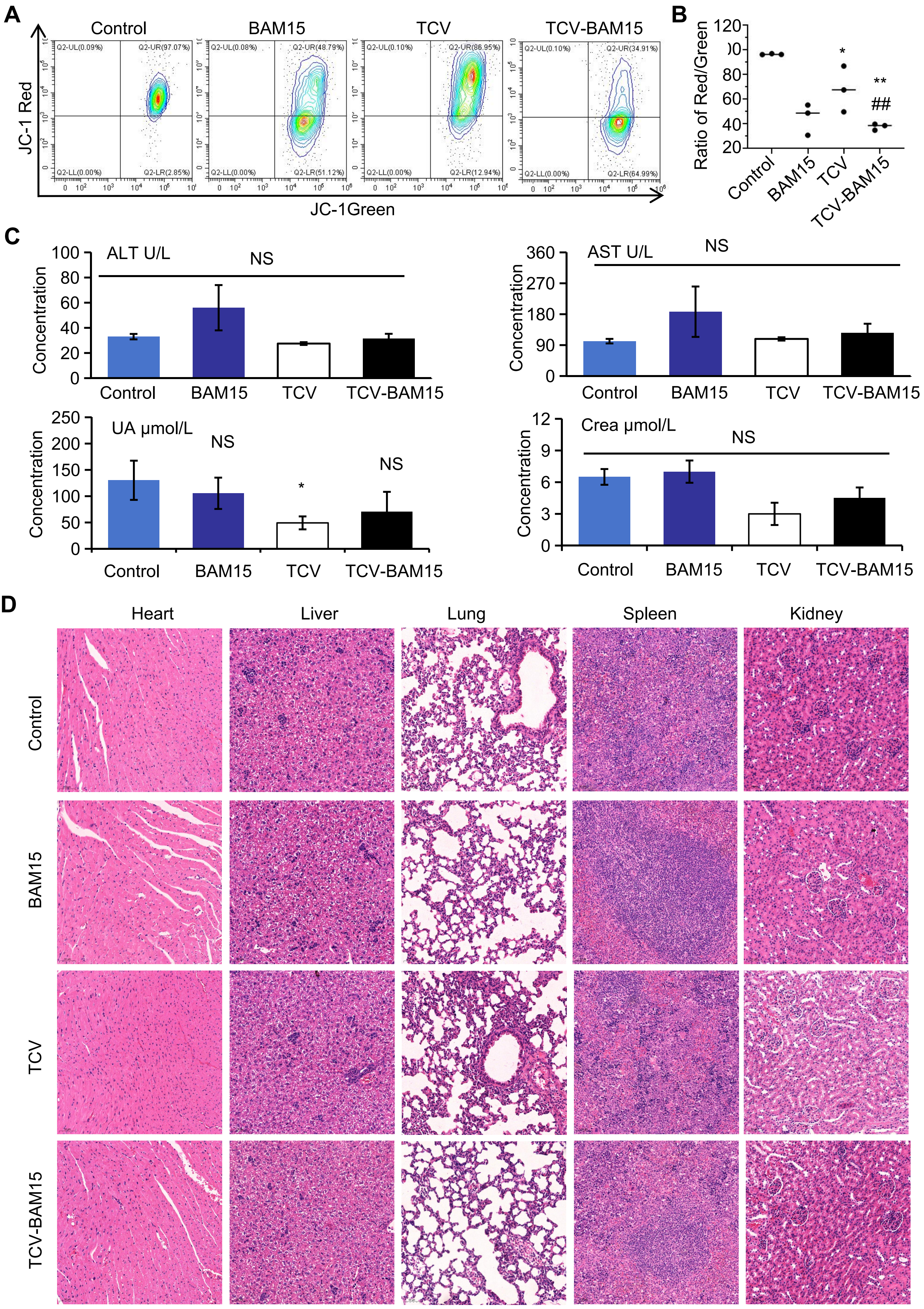
A
Control
BAM15
TCV
TCV-BAM15
JC-1 Red
JC-1Green
Q2-UL(0.09%)
Q2-UR(97.07%)
Q2-LL(0.00%)
Q2-LR(2.85%)
Q2-UL(0.08%)
Q2-UR(48.79%)
Q2-LL(0.00%)
Q2-LR(51.12%)
Q2-UL(0.10%)
Q2-UR(86.95%)
Q2-LL(0.00%)
Q2-LR(12.94%)
Q2-UL(0.10%)
Q2-UR(34.91%)
Q2-LL(0.00%)
Q2-LR(64.99%)
B
Ratio of Red/Green
120
80
60
40
20
*
**
##
Control
BAM15
TCV
TCV-BAM15
C
ALT U/L
NS
Concentration
100
80
60
40
20
0
Control
BAM15
TCV
TCV-BAM15
AST U/L
NS
360
270
180
90
0
UA μmol/L
NS
*
250
200
150
100
50
Crea μmol/L
12
9
6
3
D
Heart
Liver
Lung
Spleen
Kidney

Table 1: Primer sequences used for QPCR.

Gene name	Forward primer	Reverse primer
PD-1	CAGACTGAAAAACAGGCCGC	TGGAAGTCATGCCTGTTGGG
LAG3	AATCCTTCGGGTTACCTGGC	ACAGATTGTTCAGGGGACGC
Tigit	CTGAGGAGTCTCTCATCCCCT	TTCCTGTGGGTCAGCATAGTC
IL-15 F	GCTCTACCTTGCAAACAGCAC	TTCTCCTCCAGCTCCTCACA
Cyclophilin A	CAAATGCTGGACCAAACACAA	GCCATCCAGCCATTCAGTCT
CPT1a	CACAACAACGGCAGAGCAGAG	ACACCACATAGAGGCAGAAGAGG
FABP5	CTAGGAGTAGGACTGGCTCTTAG	GTCGTCTTCACTGTGCTCTCG
LDH	GTGGTGGTGACGGCAGGAG	GCTGTACTTGACGATCTGAGGAAT
6Pgd	GGAGTCAGTGGTGGTGAGGAAG	GCTTGGAAGATCGCCTTGATGTG
HK1	GCAAGCAGACGAGCCTAGATTG	TGACCACAGCCACCACATCC
HK2	ATCGGCGTGGATGGCTCTG	AGCGGAGGAAGCGGACATC

Figure 1J

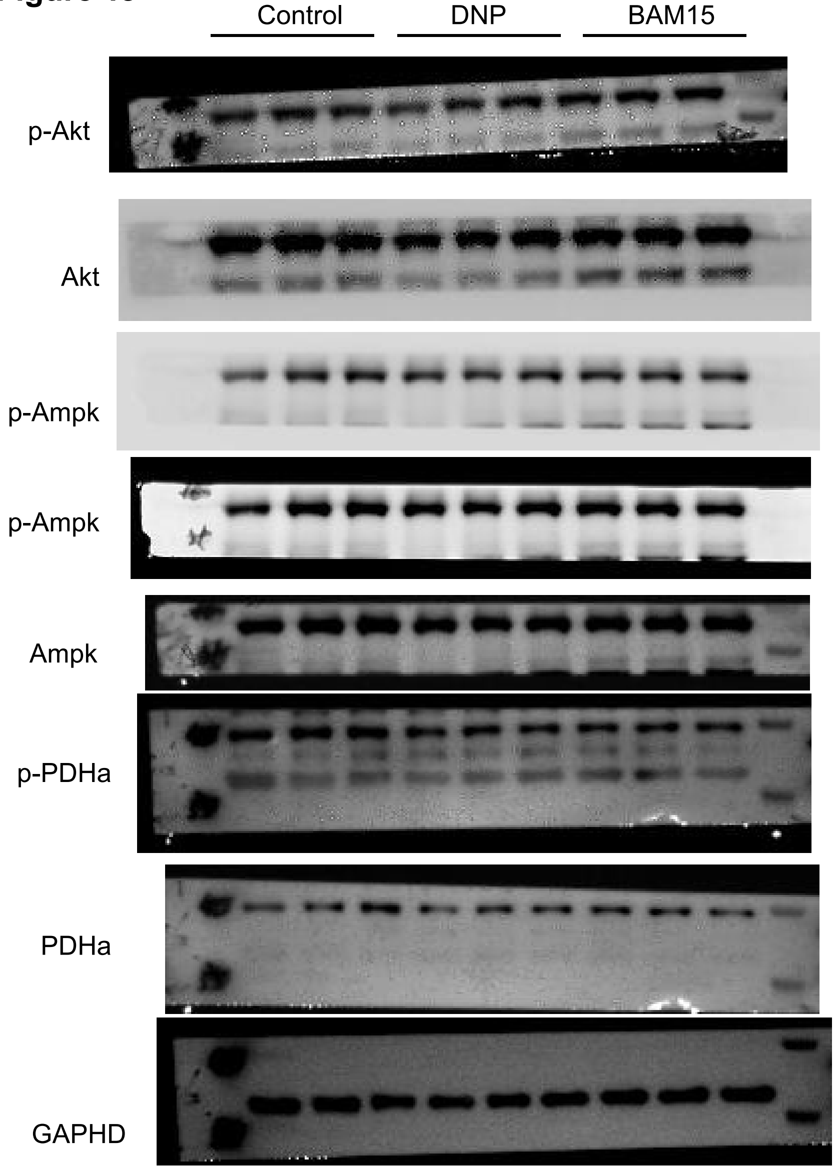

Figure 1L

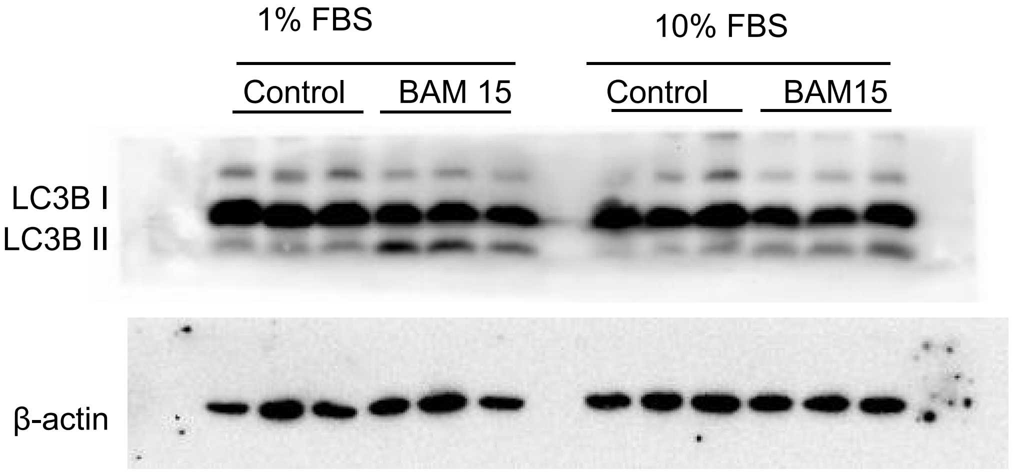

Figure 2E

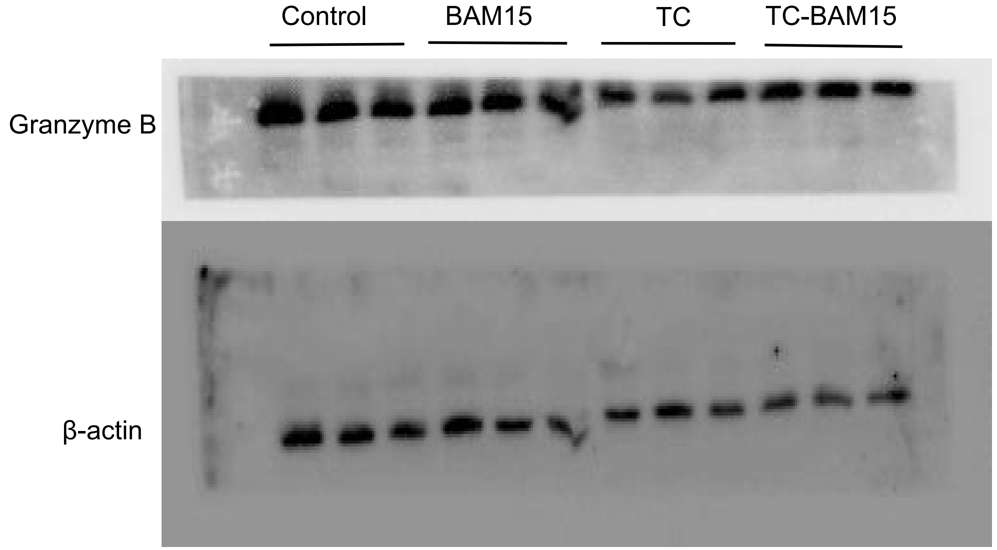

Figure 3H

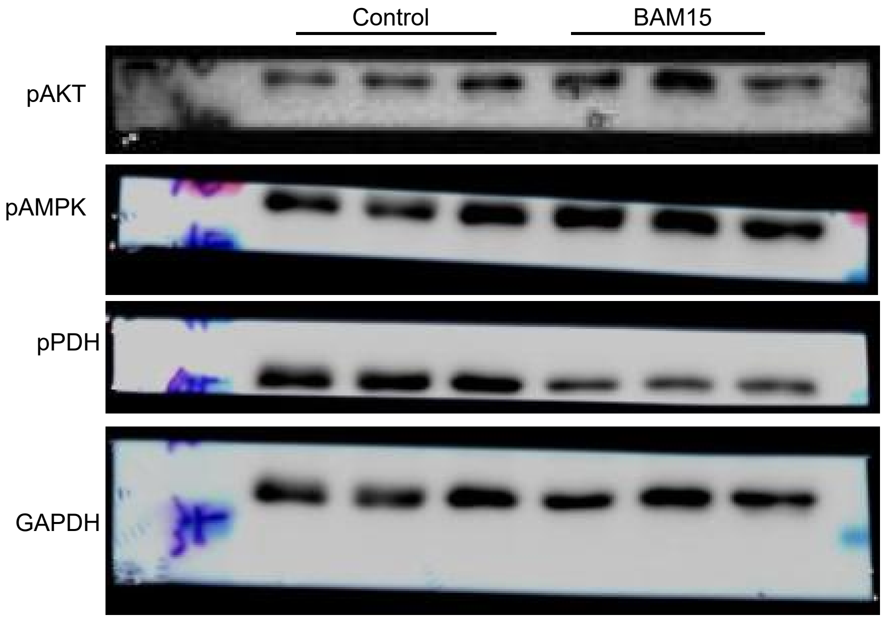

Figure 5I

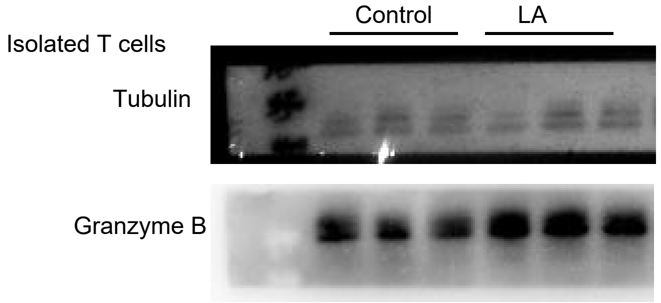

Figure 5J

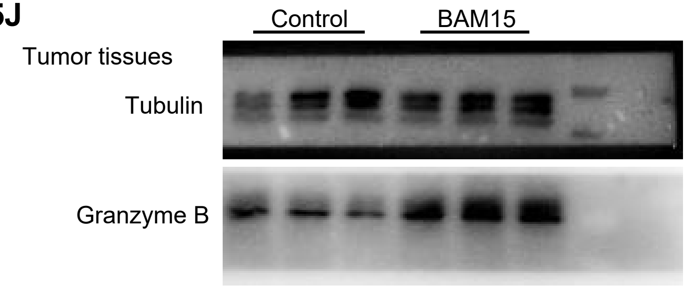

Figure 7H

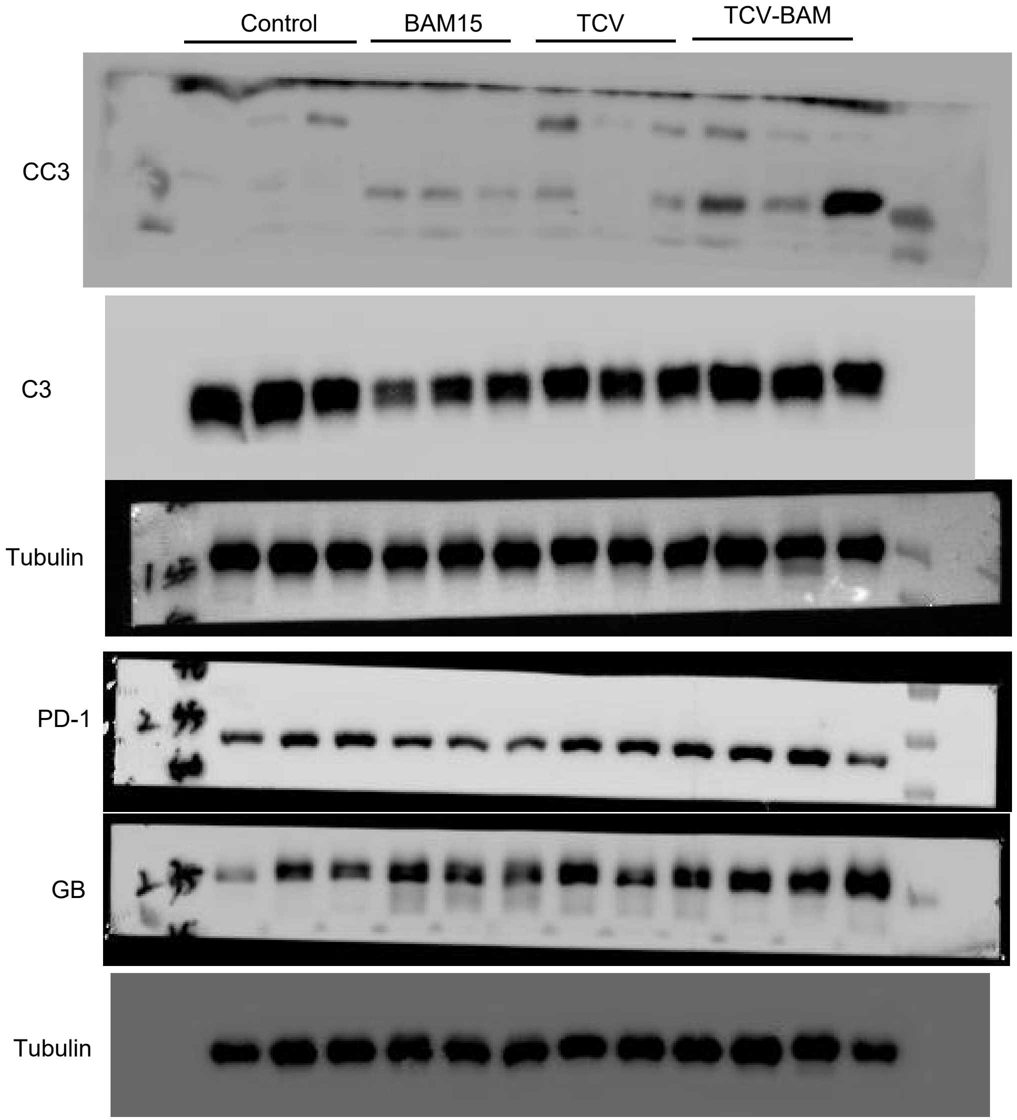

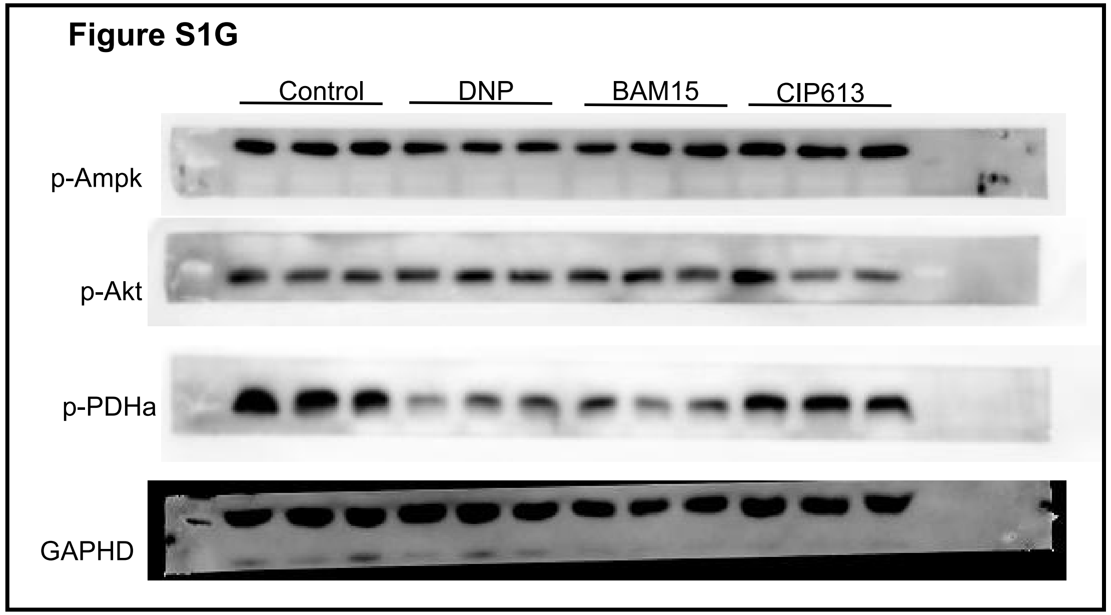
Figure S1G
Control
DNP
BAM15
CIP613
p-Ampk
p-Akt
p-PDHa
GAPHD

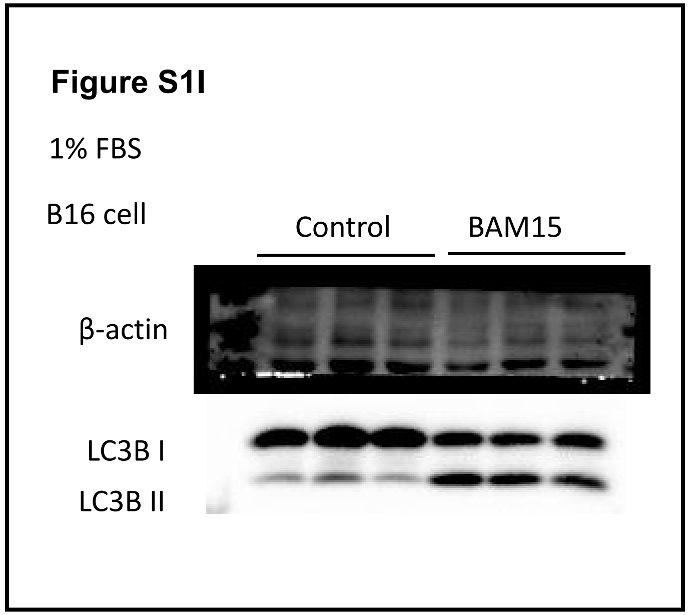
Figure S1I
1% FBS
B16 cell
Control
BAM15
β-actin
LC3B I
LC3B II
